# Molecular characterisation of side population cells with cancer stem cell-like characteristics in small-cell lung cancer

**DOI:** 10.1038/sj.bjc.6605668

**Published:** 2010-04-27

**Authors:** C D Salcido, A Larochelle, B J Taylor, C E Dunbar, L Varticovski

**Affiliations:** 1 Laboratory of Human Carcinogenesis, Center for Cancer Research, NCI, NIH, Bethesda, MD 20892, USA; 2 Albert Einstein College of Medicine, Bronx, NY 10461, USA; 3 Hematology Branch, NHLBI, Bethesda, MD, 20892, USA; 4 Flow Cytometry Core Facility, Center for Cancer Research, NCI, NIH, Bethesda, MD 20892, USA

**Keywords:** angiogenesis, drug resistance, gene expression, xenograft

## Abstract

**Background:**

Side population (SP) fraction cells, identified by efflux of Hoechst dye, are present in virtually all normal and malignant tissues. The relationship between SP cells, drug resistance and cancer stem cells is poorly understood. Small-cell lung cancer (SCLC) is a highly aggressive human tumour with a 5-year survival rate of <10%. These features suggest enrichment in cancer stem cells.

**Methods and results:**

We examined several SCLC cell lines and found that they contain a consistent SP fraction that comprises <1% of the bulk population. Side population cells have higher proliferative capacity *in vitro*, efficient self-renewal and reduced cell surface expression of neuronal differentiation markers, CD56 and CD90, as compared with non-SP cells. Previous reports indicated that several thousand SP cells from non-small-cell lung cancer are required to form tumours in mice. In contrast, as few as 50 SP cells from H146 and H526 SCLC cell lines rapidly reconstituted tumours. Whereas non-SP cells formed fewer and slower-growing tumours, SP cells over-expressed many genes associated with cancer stem cell and drug resistance: *ABCG2, FGF1, IGF1, MYC, SOX1/2, WNT1*, as well as genes involved in angiogenesis, Notch and Hedgehog pathways.

**Conclusions:**

Side population cells from SCLC are highly enriched in tumourigenic cells and are characterised by a specific stem cell-associated gene expression signature. This gene signature may be used for development of targeted therapies for this rapidly fatal tumour.

Small-cell lung cancer (SCLC) is the most devastating type of human lung cancer. In spite of recent decline in frequency, it still accounts for approximately 12% of the 220 000 new lung cancer cases projected for 2009 (Jemal *et al*, 2009). These patients usually present with disseminated disease to many organs, including the brain, and their initial response to therapy is rapidly followed by a relapse with drug-resistant disease. Small-cell lung cancer is a neuroendocrine tumour composed of cells capable of differentiation into neuronal and endocrine lineages, thus bridging two unrelated organ systems. It has high proliferative capacity, with a doubling time estimated to be as fast as 38 days in humans (Sone *et al*, 2007). The aggressive nature of SCLC combined with the capacity for differentiation into multiple lineages and the development of resistant disease suggest that this tumour may be enriched in cells with cancer stem cell-like characteristics. There is no previous information on the nature of cancer stem-like cells in small-cell lung cancer.

The existence of cancer stem cells was proposed over 30 years ago (Hamburger and Salmon, 1977) and has been fully established for haematological malignancies (Kondo *et al*, 2003; Wang and Dick, 2005). However, the origin and biology of cancer stem cells from solid tumours is still actively debated. As cancer is believed to be clonal in origin, it has been suggested that cancer-initiating cells undergo processes analogous to self-renewal and differentiation of normal stem cells, which are characterised by the ability to reconstitute the entire spectrum of cells by asymmetric division (Al Hajj and Clarke, 2004; Caussinus and Gonzalez, 2005; Hadnagy *et al*, 2006; Patrawala *et al*, 2006). Thus, similar to normal adult stem cells, cancer-initiating or cancer stem-like cells comprise only a fraction of the total tumour cell population. A recent analysis of many cell lines and clinical samples from breast, prostate and other tumours has indicated that they contain a small fraction of cells identified by a variety of cell surface markers, expression of stem cell genes, reconstitution of tumours *in vivo* and enhanced drug resistance (Sales *et al*, 2007). The frequency of tumour-initiating cells varies from 27 to 100% in highly tumourigenic, haematopoietic and melanoma primary tumours and in some cell lines (Kelly *et al*, 2007; Quintana *et al*, 2008), but it is reported to be less than 1% for most solid tumours. These differences have important clinical implications and highlight the importance of continuing the search for commonality among cancer stem cells from individual tumour types.

The ability to exclude Hoechst dye as defined by side population (SP) fraction was initially described in normal haematopoietic cells (Goodell *et al*, 1996), but was subsequently found to be present in haematopoietic malignancies and solid tumours (Hadnagy *et al*, 2006). Side population cells comprise less than 0.1% of the whole bone marrow cells and are enriched in drug-resistant haematopoietic stem cells (Sales *et al*, 2007). Several important stem cell features have been associated with the SP fraction cells: first, the ability to exclude Hoechst dye has been associated with higher expression of drug transporters, primarily of the ABC transporters family, such as ABCB1 (MDR1) and ABCG2 (BCRP), which are capable of extrusion of the dye from the cell (Goodell *et al*, 1996; Kim *et al*, 2002). Second, expression of ABC transporters is associated with drug resistance that is characteristic of stem cells from normal and malignant tissues (Szakacs *et al*, 2006). Third, the SP fraction cells are enriched in cells capable of self-renewal and differentiation with reconstitution of the original cell population (Al Hajj and Clarke, 2004; Hadnagy *et al*, 2006). Therefore, they may have a pivotal role in normal development and stem cell biology. These fundamental characteristics of normal stem cells have been applied to cells isolated from SP fraction in multiple tumours (Hadnagy *et al*, 2006; Ho *et al*, 2007), but have not been characterised in SCLC.

Owing to an aggressive clinical course and disseminated disease at presentation, patients with SCLC almost never undergo tumour resection. The diagnosis is virtually always performed on a scant material obtained by thin needle biopsy, and therapy is initiated immediately after the sample collection. Thus, there is no access to tissue samples for analysis of stem cell population in this disease. However, SCLC cells readily grow *in vitro* and numerous cell lines have been established from patients with SCLC. In contrast to other solid tumors, most SCLC cell lines do not require attachment, grow as clusters in suspension and are highly tumourigenic. Thus, studies on SCLC cell lines can be uniquely suited for examination of the cancer stem cell population. We found that SP fraction cells are substantially enriched in tumour-initiating or cancer stem-like cells that can be defined by functional analysis and by expression of cancer stem cell and drug resistance genes.

## Materials and methods

### Cells

The human SCLC cell lines NCI-H82, H146 and H526, as well as the non-small-cell lung cancer lines A549 and H460, were obtained from ATCC and maintained at 0.5 × 10^6^–2 × 10^6^ cells ml^−1^ in complete media consisting of RPMI 1640 supplemented with 10% fetal bovine serum (Lonza, Corp, NY, USA), glutamine and 1% penicillin–streptomycin (Invitrogen, Carlsbad, CA, USA) in a humidified 37^o^C incubator with 5% CO_2_.

### SP fraction *in vitro* analysis by proliferation and limiting dilution

Cells were labelled with Hoechst 33342 dye (Molecular Probes, Invitrogen) according to Goodell *et al*, (1996) with minor modifications. Briefly, cells were suspended in pre-warmed RPMI-1640 containing 2% FBS and 2 mM HEPES (HBSS) at 1 × 10^6^ cells ml^−1^ for 20 min and incubated for an additional 90 min at 37°C in a shaking bath with 5 *μ*g ml^−1^ (8.1 *μ*M) Hoechst 33342 dye. Control cells were incubated with 50 *μ*M Verapamil (Sigma, St Louis, MO, USA) for 15 min at 37°C before Hoechst dye addition. Cells were placed immediately on ice, washed and resuspended in cold HBSS containing 1% BSA. After gating viable cells identified by 7-AAD at 20 *μ*g ml^−1^ (BD Pharmingen, Lexington, KY, USA), SP and non-SP fractions were gated separately and analysed using an LSRII flow cytometer (BD Biosciences, San Jose, CA, USA).

For limiting dilution assay *in vitro*, sorted cells were plated 50 *μ*l per well in 96-well plates in complete media. The media was added biweekly for the following 2 weeks and colonies were scored. Proliferative capacity was determined by plating <2 cells per well and the media was added as above. The number of wells that contained colonies was scored at the end of 3 weeks.

### Cell surface immunophenotyping

Immunophenotyping was carried out using conjugated monoclonal human antibodies reactive to CD24, 34, 44, 45, 56, 87, 90, 117, 133, CXCR4, ABCB1 and ABCG2 (BD Pharmingen). The staining was performed in the dark at 4°C for 30 min. Isotype control antibodies and live unstained cells were used to establish gating parameters for positive cells. Mean fluorescence intensity (MFI) was determined using FlowJo Software (NCI license, Asland, OR, USA).

### Stem cell gene expression by focused array

The mRNA levels of 84 genes associated with stem cell biology were examined simultaneously using human Stem Cell RT^2^ profiler arrays (SuperArray Bioscience, Frederick, MD, USA) according to the manufacturer's instructions. Briefly, total RNA was isolated using TRIzol (Invitrogen) followed by spin-column purification with RNeasy extraction kit (Qiagen, Valencia, CA, USA) in the presence of DNAse. Total RNA (250 ng) was reverse transcribed using the First Strand Synthesis Kit and cDNA was subjected to real-time PCR using SYBR green/ROX Master Mix on a 7500 Real-Time PCR System (Applied Biosystems, Foster City, CA, USA). Values obtained for the threshold cycle (Ct) for each gene were normalised using the average of values of housekeeping genes. The difference (ΔCt) between SP and non-SP RNA values was determined by ΔCt=Ct(SP)–Ct(non-SP) and fold change by fold change=2(−ΔCt).

### Validation of gene expression by quantitative-PCR

Quantitative-PCR (Q-PCR) analysis was carried out using TaqMan probes (Applied Biosystems) according to the manufacturer's instructions, in a 10 *μ*l final reaction volume using 384-well microtitre plates. One microgram of total RNA was reverse transcribed into cDNA using the Single-Strand cDNA Synthesis Kit (Stratagene, La Jolla, CA, USA) and analyzed using ABI-7900 (Applied Biosystems, Foster City, CA, USA). Specific primers for Q-PCR of *GAPDH, RPL13A and ACTB* (housekeeping genes) and the additional genes of interest (Figure 5) were designed using Applied Biosystems Assay-by-Design primer design software or were purchased as Assays-on-Demand from Applied Biosystems. Quantification of each mRNA was achieved by normalising the sample values to the universal Stratagene (Novoradovskaya) reference from human liver. The samples were normalised to human GAPDH, RPL13A and ACTB individually, as well as to the average of all three endogenous controls. The expression level of each gene in the SP was compared with the corresponding level in non-SP fractions in triplicates from three independent experiments.

### *In vivo* tumour formation

All studies were conducted in an AAALAC-accredited facility, in compliance with the US Public Health Service guidelines for the care and use of animals in research under protocols approved by the ACUC. Naive male 6–8-week-old NOD/*SCID* mice from the NCI Animal Production Program (NCI-Frederick, Frederick, MD, USA) or Jackson Laboratories (Bar Harbor, ME, USA) were used as tumour transplant recipients. For *in vivo* tumour formation, growing cells sorted from the SP and non-SP fractions or the bulk population, diluted in PBS, were mixed with 50 *μ*l Matrigel (BD Bioscences) and injected subcutaneously. Tumour growth was measured biweekly and the weights (in milligrams) were calculated using the formula for a prolate ellipsoid and assuming a specific gravity of 1.0 g cm^−3^ using the formula *L* × *W*^2^ × 0.5 (Plowman *et al*, 1997). For isolation of cells from xenografts, freshly isolated tumours were made into single-cell suspension as described (Varticovski *et al*, 2007).

### Statistical methods

Differences were determined using two-tailed non-paired *t*-test or by two-sided Fisher's exact test, as indicated in the text.

## Results

### SCLC cell lines contain a side population that excludes Hoechst dye (SP fraction)

We examined H82, H146 and H526 SCLC cell lines by flow cytometry of live cells for exclusion of Hoechst dye, and defined the gating of these cells by disappearance of this fraction in cells pre-incubated with the transporter inhibitor, Verapamil. All SCLC cell lines examined contained less than 1% in SP fraction. H146 cells contained 0.8±0.1 and H526 had 0.9±0.4% of SP cells in the total cell population (Supplementary Figure 1). Supplementary Figures 1A and B also illustrate the SP gating for H146 and H526 cells, wherein the gating is defined by pretreatment with Verapamil. Mouse bone marrow cells with an SP fraction of 0.1% were used as a positive control and are shown on the lower panel. As previously reported (Ho *et al*, 2007), the non-small-cell lung cancer cell lines A549 and H460 have a higher SP fraction (2–4%) (Supplementary Figure 2). We also tested cells isolated from SP and non-SP fractions for viability after exposure to Hoechst dye at concentrations used in these studies using MTT dye staining (Wright *et al*, 2008), and found no impairment of cell viability.

Previous studies indicated that expression of ABC transporters was associated with the SP fraction and expression of ABCG2 was described to be associated with normal and malignant stem cells (Farnie and Clarke, 2007; Ho *et al*, 2007). Although there was a small increase in ABCB1 and ABCG2 transporters in SP fraction cells in some experiments (Supplementary Figure 2B), we found no statistically significant differences on repeated experiments and none of the other SCLC cell lines examined had significant cell surface expression of ABCB1 or ABCG2 (data not shown).

**Figure 1:**
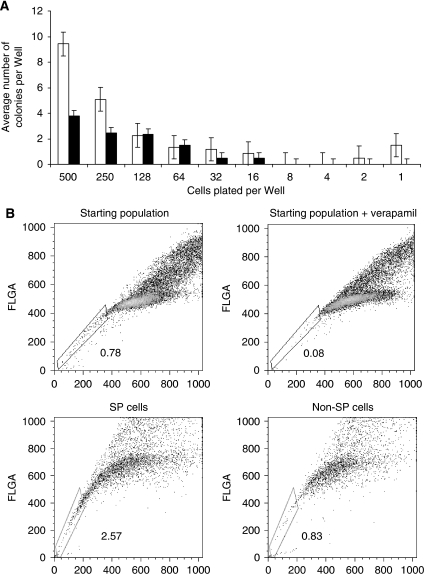
Characterisation of cells from SP and non-SP fractions. (**A**) Side population cells have higher proliferative capacity *in vitro*. H146 cells were sorted from SP and non-SP populations and plated by limiting dilution in six-tuplicate wells. The number of growing colonies at the end of 3 weeks was scored and data (expressed as mean number of colonies per well) were averaged from two independent experiments±s.e. Open bars: SP cells; solid bars: non-SP cells. (**B**) Cells sorted from SP fraction repopulate the original population. Side population and non-SP cells were sorted according to Verapamil gating (upper panels) and cultured for 17 days (lower panels).

To determine the differences in proliferation, cells were isolated by sorting from the SP and non-SP fractions and plated by limiting dilution from 500 to 1 cell per well in 96-well plates in replicates of six wells in complete growth media. Cells isolated from the SP fraction were more likely to survive and form viable colonies at the end of 3 weeks. We did not see appreciable differences in colony size formed by SP and non-SP cells, but noted that SP fraction cells grew colonies even when plated at very low cell density number (Figure 1A).

### Self-renewal capacity of SP and non-SP cells

To determine differences in self-renewal capacity of cells from SP and non-SP fractions, 10 000 cells were isolated from each fraction and cultured for 2–3 weeks until the total cell number was increased to approximately one million cells, a number required for adequate SP analysis. Of 100% SP cells sorted at the beginning of the experiment, only 3% remained after 17 days in culture (Figure 1B). These data indicate that the absolute number of SP cells was retained in the expanded population, and the overall population lost the ability to efflux Hoechst dye. Interestingly, non-SP cells also acquired a small SP fraction within 17 days. Extending the period of observation to 6 weeks resulted in reconstitution of the original populations observed in unsorted cells, with <1% of cells in SP fraction (data not shown). Considering that the doubling time for H146 cells is approximately 30–36 h, the SP fraction cells preserved self-renewal without appreciable expansion *in vitro*. These observations also suggest that the non-SP cells acquire SP function or that the gating cells solely based on flow cytometry do not allow absolute separation of these cells. These data confirm that Hoechst dye labelling and separation by flow cytometry are not exclusive.

### Expression of cell surface markers in SP and non-SP cells

**Figure 2:**
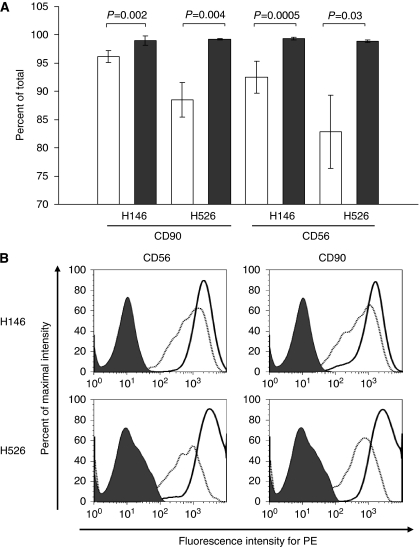
Expression of differentiation markers is decreased in SP fraction. (**A**) Percent cell surface expression of CD56 and CD90 within the SP and non-SP cells in H146 and H526 cells. Solid bars: SP cells; open bars: non-SP cells±s.d. The differences between each pair are statistically significant as determined by the two-tailed Fisher ’s *t*-test, indicated by P values above. (**B**) Cell surface expression in H146 and H526 cells for each marker using PE-labelled antibodies. The dashed line represents cells in SP fraction, and the solid line shows cells in non-SP fraction. The filled area represents unstained cells.

To further define the cells within the SP fraction, we used multiple cell surface markers described in association with neuroendocrine tumours and cancer stem cells (see Materials and Methods). The analysis of three SCLC lines (H82, H146 and H526) revealed that these cells differ significantly in expression of cell surface markers. Only a few markers such as CD56 (N-CAM, neural cell adhesion molecule 1 isoform, a hallmark of SCLC), CD90 (Thy-1, a neuronal and mesenchymal stem cell marker), CXCR4 (metastasis-associated G-protein receptor) and CD44 (associated with breast and other CSCs) were expressed in all cell lines, whereas expression of CD133, associated with cancer stem cells in many other tumour types (Mizrak *et al*, 2008), including non-small-cell lung cancer (Eramo *et al*, 2008), varied from nearly 100% positive in H82 to 20% in H146 and was non-detectable in H526 cells. However, we found that the overall percent of cells positive for CD56 was significantly lower in SP as compared with non-SP cells in all cell lines examined, as shown for the H146 and H526 cells in Figure 2. Although the decrease in CD90 cell surface expression in the SP fraction in H146 cells was small, it was statistically significant and substantially different in H526 and H82 (Figure 2, data not shown). In addition, measurements of MFI were lower in SP cells (Figure 2B and Supplementary Table I), indicating lower cell surface protein expression per cell. No statistically significant changes in other cell surface proteins, including ABCG2, CD133 and CD44, were observed in these cells.

### SP fraction cells are enriched in tumour-initiating cells *in vivo*

**Figure 3:**
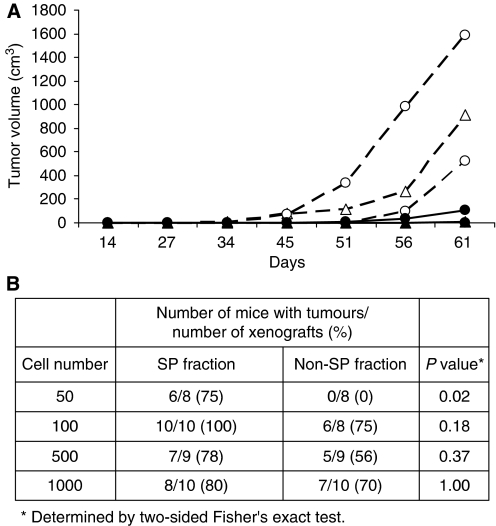
Tumorigenicity of SP and non-SP cells *in vivo*. (**A**) In total, 50 (circles), 100 (triangles) and 500 (squares) H146 cells were implanted in triplicate injections and tumour growth rates of SP cells (dashed lines, open symbols) and non-SP cells (solid lines, closed symbols) were monitored biweekly. The lines represent the average tumour volume calculated from one experiment, as indicated in Materials and Methods. (**B**) Summary of H146 tumours formed following implantation of the indicated cell numbers from SP and non-SP cells from four independent experiments. Small tumours that were only palpable (<5 mm^2^) were scored as positive.

We tested tumour reconstitution *in vivo* using a limiting dilution of cells sorted from the SP and non-SP fractions. We did not use additional markers because we did not find additional markers for positive selection that are common to the SCLC cell lines. As seen in Figure 3 and Supplementary Figure 3, as few as 50–100 SP cells from H146 and H526 cells, respectively, were sufficient to rapidly reconstitute the tumours, whereas mice that received 50–100 non-SP cells did not develop tumours, or had only palpable tumours in the same time frame (less than 5 mm^3^). All mice implanted with 500 SP cells rapidly developed tumours that had to be removed within 3–4 weeks, whereas mice that received non-SP cells (5 of 9) had only small tumours in that period of time. These non-SP tumours grew considerably slower and reached 300–500 cm^2^ size after additional 30 days of observation. Thus, SP fraction cells are considerably more efficient in tumour reconstitution *in vivo*. The tables in Figure 3 and Supplementary Figure 3 summarise the results of multiple independent experiments showing the frequency of tumour formation by SP and non-SP cells obtained from H146 and H526 cell lines, respectively. The major differences in tumour-initiating frequency between SP and non-SP cells are apparent at low numbers of injected cells (50–100). We conclude that, in spite of minor differences in growth rates *in vitro*, the SP fraction cells are significantly enriched in tumour-initiating cells. Analysis of SP fraction from xenografts formed by SP fraction cells showed that these tumours returned to the original proportion of SP cells, with a very small (<1%) SP fraction. This is consistent with our *in vitro* self-renewal data and observations from other investigators in other tumour types (Wright *et al*, 2008; Eramo *et al*, 2008).

**Figure 4:**
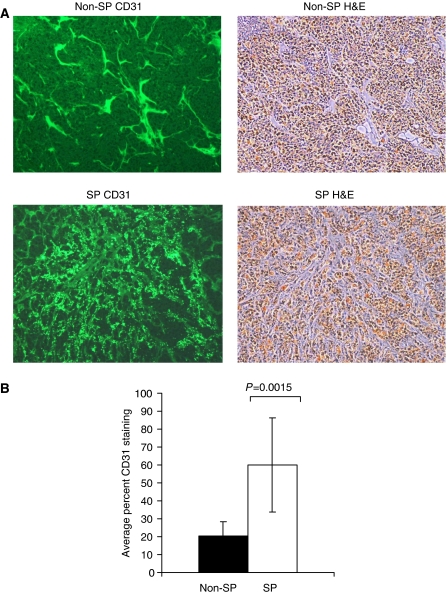
Tumours arising from SP fraction cells a have higher degree of angiogenesis. (**A**) Microvessel staining using mouse anti-CD31 antibodies of one representative field from tumours generated from 500 non-SP (upper panel) and 500 SP (lower panel) H146 cells. H&E staining for each tumour is showed on the right. (**B**) Computer-generated quantitative analysis of anti-CD31 staining fluorescence intensity from one representative tumour. Non-SP (filled bars) and SP (open bars). Bars represent s.d. from 9–10 computer-generated random fields from three slides from one representative tumour. The measurements are from one of two individual tumours±s.d.

The H&E stain was performed on tumours formed by equal numbers of SP and non-SP cells when they reached comparable tumour size. This analysis revealed the features and characteristics of SCLC tumours with hyperchromatic nuclei, abundant cytoplasm, finger-like projections and frequent mitotic figures with no significant differences in morphology between SP and non-SP formed tumours (Supplementary Figure 4). We also found no evidence of metastasis into lung, liver and other major organs by gross pathological examination. Staining for Ki67 also did not reveal significant differences. Although the vascular structure of tumours formed by SP cells was somewhat more pronounced, it was not readily apparent on all sections and all tumours examined. However, analysis of vascular staining using mouse endothelial cell marker, CD31, showed that SP tumours had an abundant angiogenic response, were punctate and tortuous staining of microvessels that was not apparent in tumours formed by non-SP cells (Figure 4). Quantitative analysis of fluorescence intensity using computer-generated random fields from several tumours confirmed a significant increase in the overall intensity of staining in tumours formed by SP as compared with non-SP cells (Figure 4, lower panel).

### Analysis and validation of gene expression in SP and non-SP cells

*Analysis of stem cell genes.* To examine the expression of stem cell genes from cells in SP and non-SP fractions, real-time quantitative RT–PCR on 84 genes was carried out using commercially available Stem Cell RT^2^ Profiler_TM_ PCR Focused Array (SuperArray Bioscience Corp, Frederick, MD, USA) by using two biological replicates from H146 cells. The list of all 84 stem cell genes on that panel can be found at http://www.sabiosciences.com/howpcrarrayworks.php. Twenty-two genes were significantly upregulated in SP as compared with the non-SP cells (Supplementary Table II), whereas two genes, both associated with cell–cell interactions, were downregulated (*GJB1* and *PARD6A*). Overall, there were more upregulated stem cell genes in SP cells. These features are consistent with previous reports on enrichment in genes associated with growth and developmental pathways in SP cells from normal tissues (Behbod *et al*, 2006, 2006; Larderet *et al*, 2006). In addition, several of the upregulated genes, including *ABCG2, BMP1/2, FGF1, IGF1, MYC, SOX1/2, WNT1* and *NOTCH2*, have been recently associated with cancer stem cells and tumour-initiating cells in lung cancer and other tumour types, as well as with epithelial-to-mesenchymal transition in tumours with poor prognosis (Fan *et al*, 2006; Moustakas and Heldin, 2007). Interestingly, upregulation of ABCG2 transporter mRNA did not correlate with the level of cell surface protein.

**Figure 5:**
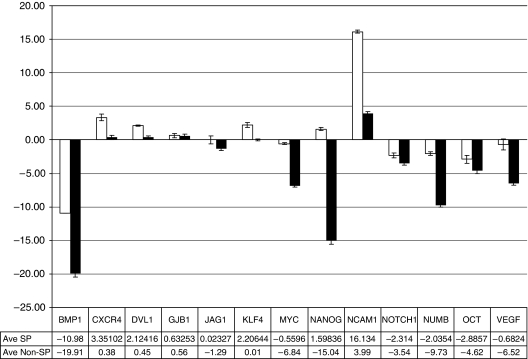
Gene expression analysis validation by quantitative RT–PCR. Gene expression for SP fraction (open bars) and Non-SP fraction (closed bars) was analysed as described in Materials and Methods. The data are normalised to a human liver as universal standard (±s.d. from triplicate samples) using three sorting experiments representing independent biological replicates. All genes shown were significantly upregulated in SP cells, except for GB1 and JAG1. The graph shows the results in a format (both numerically and graphically) showing expression of each gene in SP and non-SP fractions relative to combination of four housekeeping genes and a reference universal standard (human liver).

*Validation by quantitative RT—PCR.* Using independent quantitative RT–PCR, we validated few of the significantly changed genes as well as examined additional genes associated with tumour progression, angiogenesis and stem cell characteristics that were not included in the SuperArray panel. We used the same RNA samples as for the SuperArray and additional samples from biological replicates from three independent paired sorts of SP and non-SP cells. The values shown in Figure 5 were normalised to the three housekeeping genes and to the expression in universal standard (human liver). The increase in expression of BMP1 and MYC in SP cells was remarkably similar to the SuperArray data (Figure 5). In addition, upregulation of other stem cell and pluripotency-associated genes, *KLF4, NANOG, NUMB, OCT4* and *NOTCH1,* in SP cells became apparent by this analysis. An increase in VEGF expression in the SP fraction (Figure 5) and their xenografts (data not shown) could be responsible for the substantial angiogenic response *in vivo*. The high expression of CXCR4 in SP cells, together with the increase in its ligand CCL12 (SDF-1) detected by SuperArray analysis (Supplementary Table II), also suggests that these cells have higher migration and metastatic potential (Das *et al*, 2008).

*Correlation with protein expression.* To further validate these results, we examined cell surface protein levels for those proteins for which we had antibodies suitable for flow cytometric analysis. Although the function of CD8*β* chain is not well defined, we included this protein in the analysis as it has a role in chromatin modifications and alterations in its consensus coding sequences have been found in human breast and colorectal cancers (Sjöblom, 2006). Expression of three proteins examined (IGF1, FGF1 and CD8*β*) was increased in H146 SP cells by 1.2–4.0 fold, with a corresponding increase in MFI. Cell surface expression of CD8β and FGF1, but not that of IGF1, was also increased in the SP fraction of H526 cells (data not shown).

## Discussion

We observed novel features in a sub-population of SCLC SP fraction cells that have cancer stem cell-like characteristics. Previous studies using SP fractions from many tumours showed relative enrichment in tumour-initiating cells, but several hundred or thousand cells were required for tumour reconstitution (Al Hajj *et al*, 2003; Collins *et al*, 2005; Ponti *et al*, 2005). In studies of non-small-cell lung cancer, 1–5 × 10^3^ SP cells (Ho *et al*, 2007) or 1 × 10^4^cells that express CD133 markers (Eramo *et al*, 2008) were needed for tumour reconstitution. In contrast, we found that as few as 50–100 SP cells from SCLC lines were sufficient to reconstitute tumours in NOD/SCID mice, whereas at least 500 non-SP fraction cells were required to form tumours under the same conditions, although few of those tumours reached 300–400 cm^2^ after an additional 30 days of observation. Implantation of 50–100 non-SP cells resulted in either no tumour or formation of barely palpable tumours that did not progress during the additional 30 days of observation. A recent report showed that as little as 10 cells co-implanted with one million unselected immune cells reconstitute haematopoietic tumours in syngeneic recipients (Kelly *et al*, 2007). The differences with reports from solid tumours were attributed to use of a syngeneic model. However, studies comparing tumour growth rates of cells from transgenic Wnt1 mouse mammary tumours implanted into syngeneic, nude or SCID mice did not show appreciative differences (Varticovski *et al*, 2007; Svirshchevskaya *et al*, 2008). Thus, the high capacity of tumour reconstitution using haematopoietic tumour cells may be an intrinsic feature of haematopoietic malignancies.

In addition to a faster growth rate, the tumours that arose from SCLC SP cells showed a significant degree of neo-angiogenesis. These tumours, the corresponding SP fraction cells and tumours that arose from these cells showed upregulation of VEGF, a feature not previously reported for these cells. Side population-sorted cells also reconstituted the entire cell population within 2 week in culture, a feature consistent with efficient self-renewal, which is an essential characteristic of cancer stem cell-like cells. In addition, the SP cells overexpressed *NANOG* and *SOX2*, genes associated with self-renewal process.

As non-SP fraction cells also formed tumours, although at a significantly reduced rate and with delayed tumour growth rates, it is possible that sorting SP cells, as defined by Verapamil gating, do not permit absolute selection, and a few cells in non-SP fraction have the capacity of dye efflux. This was confirmed in the self-renewal experiments. Cells sorted from the non-SP fraction after 2 weeks in culture acquired 0.6–0.8% of cells that were able to efflux the dye. Thus, it is not possible to achieve complete separation of dye by excluding cells in a single sorting passage by flow cytometry, and the non-SP fraction is likely to contain a small percentage of cells that can also efflux the dye. Thus, the SP fraction analysis needs to be compared with other methods, including staining for ALDH. Alternatively, efflux of Hoechst dye is only one of the many functional characteristics of tumor-initiating cells. Previous reports indicate that haematopoietic stem cells are also present in the non-SP compartment (Morita *et al*, 2006), and mammary repopulating cells have also been found in the non-SP fraction. Thus, these compartments are not strictly defined in normal organs, and some overlap is expected.

These considerations prompted us to undertake an extensive search for additional cell surface and genetic markers to define further the cancer stem-like cell population in the SP fraction. In spite of the extensive search, we could not identify any common surface markers that would be enriched in the SP fraction of all SCLC cell lines we examined. However, we observed a consistent decrease in SP cells in two markers, CD56 and CD90. These cell markers represent features associated with neuronal differentiation of SCLC and other organs, and their low expression is consistent with the primitive nature of SP cells. CD56 (N-CAM, neural cell adhesion molecule 1 isoform) is a hallmark of SCLC. CD90 (Thy-1) is a neuronal and mesenchymal stem cell marker that also defines the neuronal differentiation of these neuroendocrine tumours. In contrast to our data using SCLC, cell surface expression of Thy-1 was reported to be enriched in CD133+ tumour stem cells from brain tumours (Liu *et al*, 2006; Mizrak *et al*, 2008) characterised by self-renewal, high proliferative capacity *in vitro* and tumour reconstitution *in vivo* (Al Hajj *et al*, 2003; Ponti *et al*, 2005; Sales *et al*, 2007; Eramo *et al*, 2008). However, we found no change in frequency or enrichment in any of the previously identified cell surface markers, including CD133, in the SP fraction cells in SCLC cells. This may reflect high expression of CD133 in H146 and H82 cells (20% and 90%, respectively). Thus, stem-like cells from SCLC have features similar to the haematopoietic malignancies, with enrichment in SP fraction, rather than expression of cell surface markers described for other solid tumours.

ABCG2 transporter expression is associated with drug resistance and Hoechst dye efflux (Farnie and Clarke, 2007; Ho *et al*, 2007). We did not detect an increase in ABCG2 transporter protein on cell surface. However, gene expression analysis showed upregulation of mRNA for the ABCG2 transporter. These changes were subsequently confirmed by independent RT–PCR. The previously reported rapid membrane turnover of ABC transporters may be responsible for this phenomenon (Robey *et al*, 2007).

Of significance, SP fraction cells showed upregulation of genes that werepreviously associated with normal stem cell biology and pluripotency. Similar to our observations, transcriptional profiling of SP cells from several non-malignant tissues showed that more genes were upregulated than downregulated and included genes associated with multi-drug resistance, regulation of transcription, cell signalling, Notch and Wnt pathways (Liadaki *et al*, 2005; Behbod *et al*, 2006; Larderet *et al*, 2006). Side population fraction cells examined in our studies have upregulated genes that are involved in pathways modulating stemness, including *MYC, FGF1, OCT4, KLF4, NOTCH2* and *WNT*. These data confirm that SCLC cell lines contain a population of highly undifferentiated cells with stem cell-like characteristics.

Development of agents that target the Hedgehog pathway, which is re-activated in bronchial mucosa following epithelial injury and in progenitor cells (Watkins *et al*, 2003; Vestergaard *et al*, 2006), renewed the interest in using these agents in treatment of SCLC. Recent studies showed that stem-like cells in brain tumours are selectively vulnerable to agents inhibiting the Notch pathway (Fan *et al*, 2006). The feasibility of similar approaches in SCLC remains to be established.

In summary, our findings identified a small population of SCLC cells that resides in the SP fraction and has functional and molecular features consistent with cancer stem cells. Further characterisation of their genetic signature, epigenetics and metabolic features could have direct therapeutic implications.

## Supplementary Material

Supplementary Table I

Supplementary Table II
