# Supplementary figures and images for: Molecular characterisation of side population cells with cancer stem cell-like characteristics in small-cell lung cancer
Source: Br J Cancer. 2010 Apr 27;102(11):1636–44. doi: 10.1038/sj.bjc.6605668 (PMC2883147; doi:10.1038/sj.bjc.6605668)

Supplemental Figure 1

H146

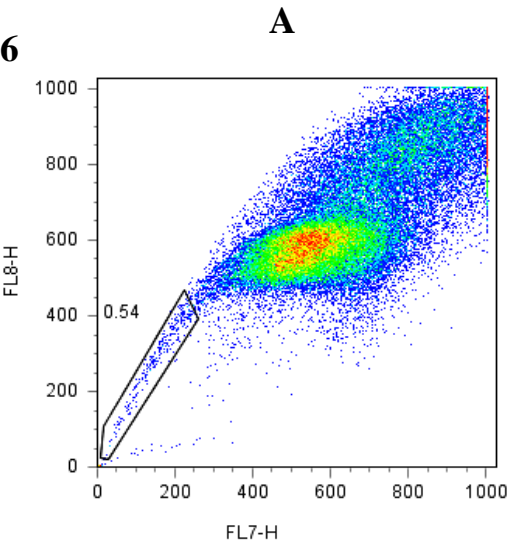

**B**

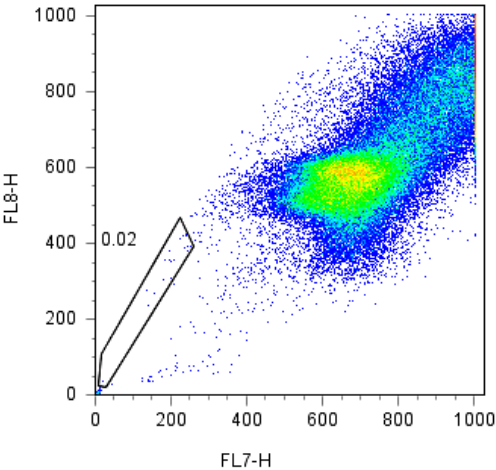

H526

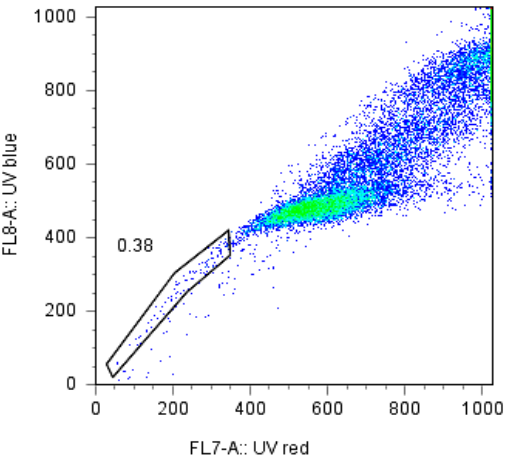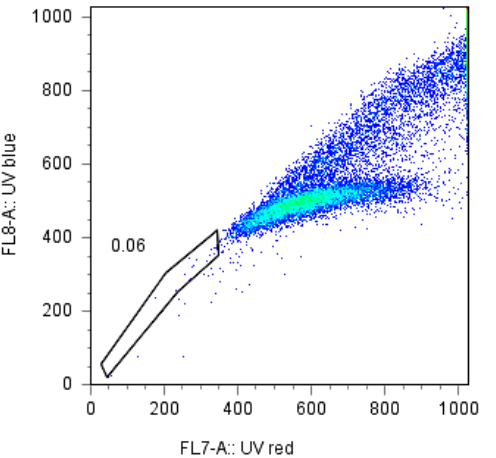

Mouse BM

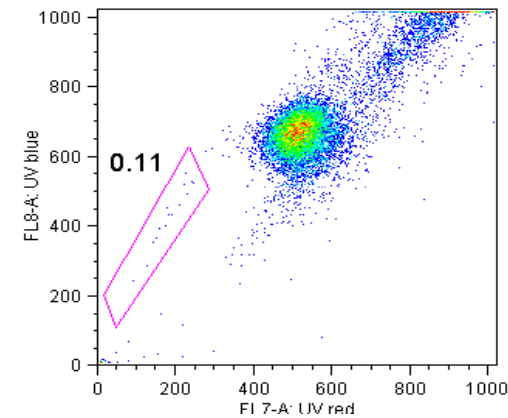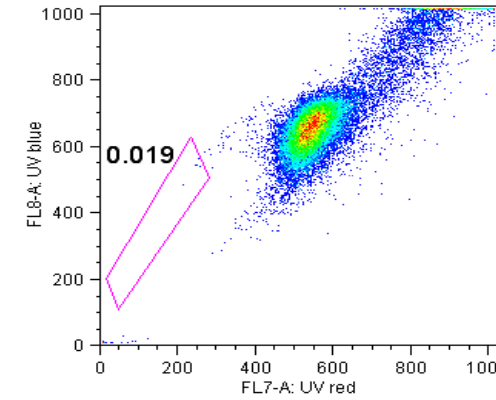

Supplement: Supplementary Figure 1 [file 6605668x1.pdf]

Supplemental Figure 2

A

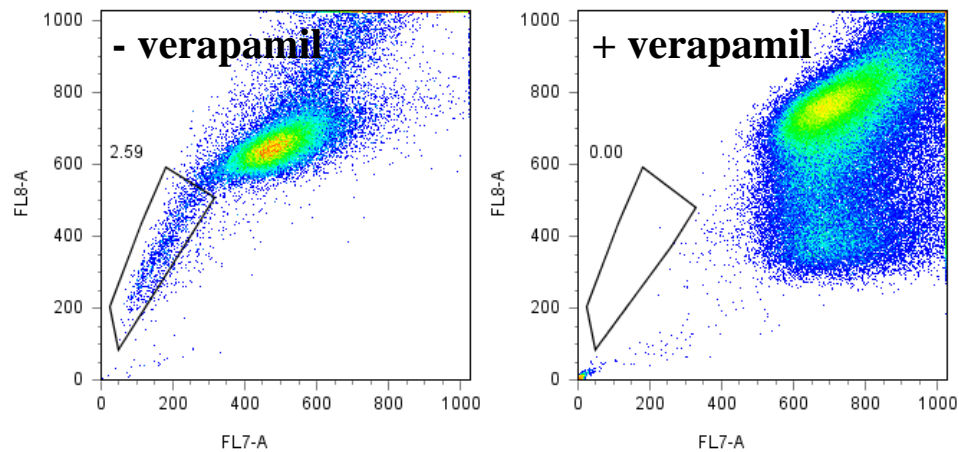

B

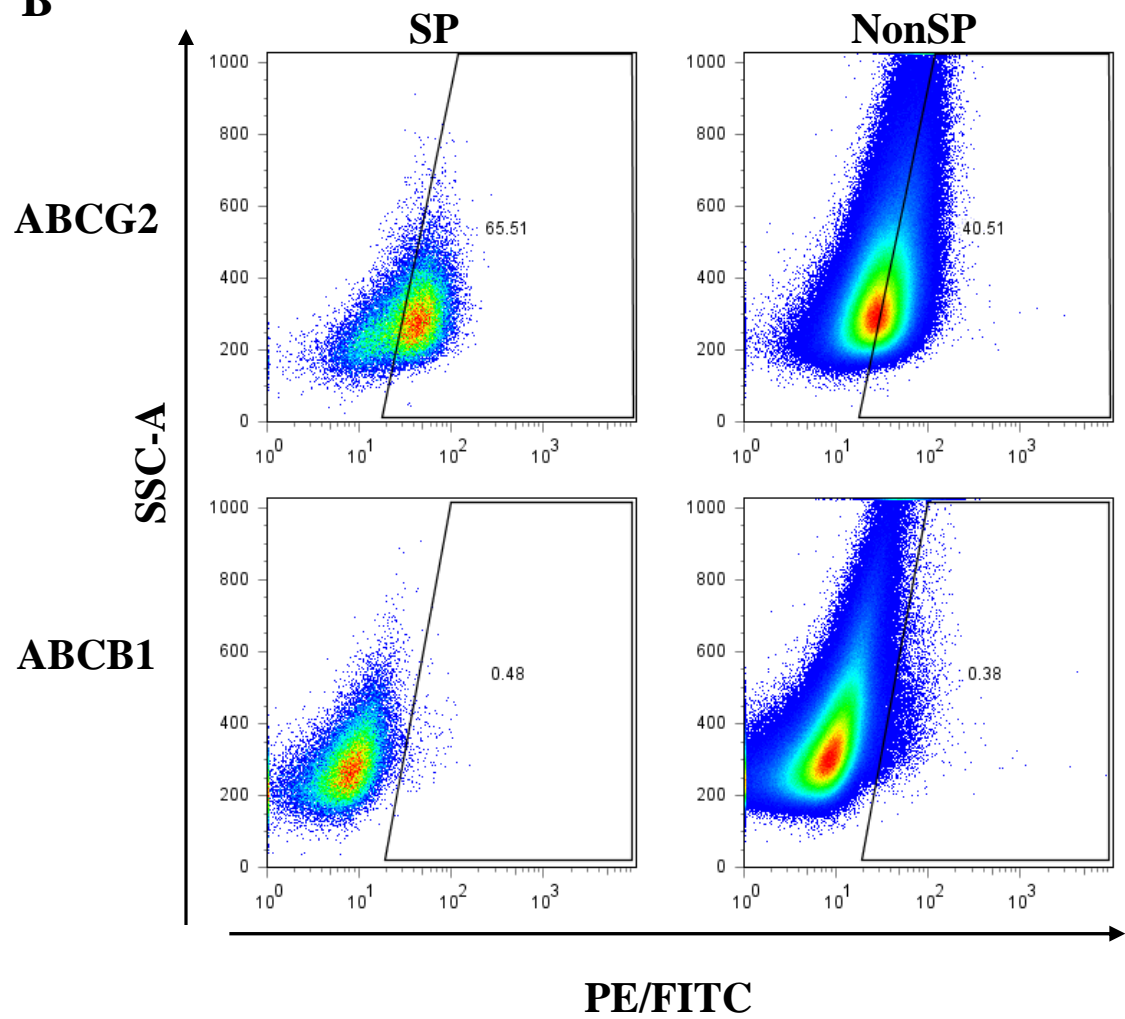

Supplement: Supplementary Figure 2 [file 6605668x2.pdf]

## Supplemental Figure 4

### H146 SP

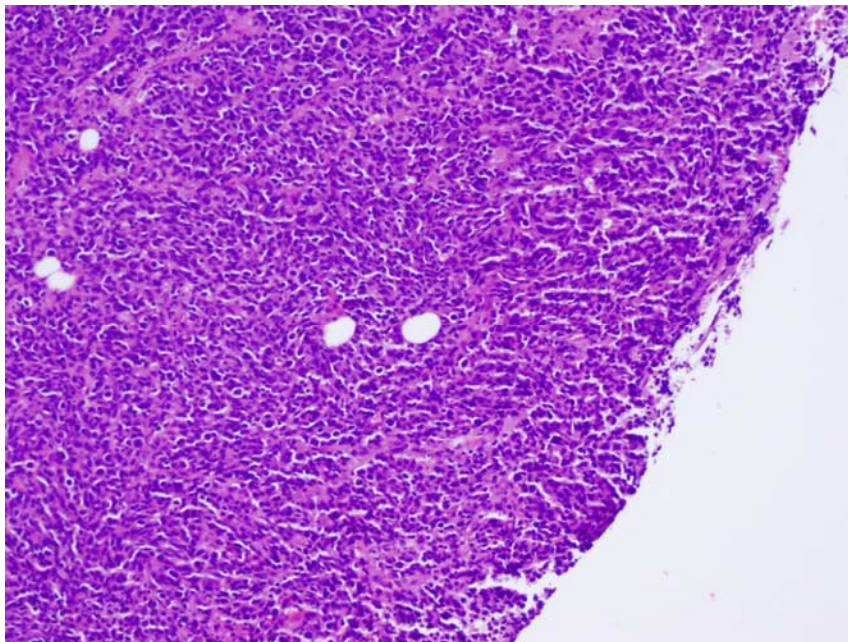

### H146 Non-SP

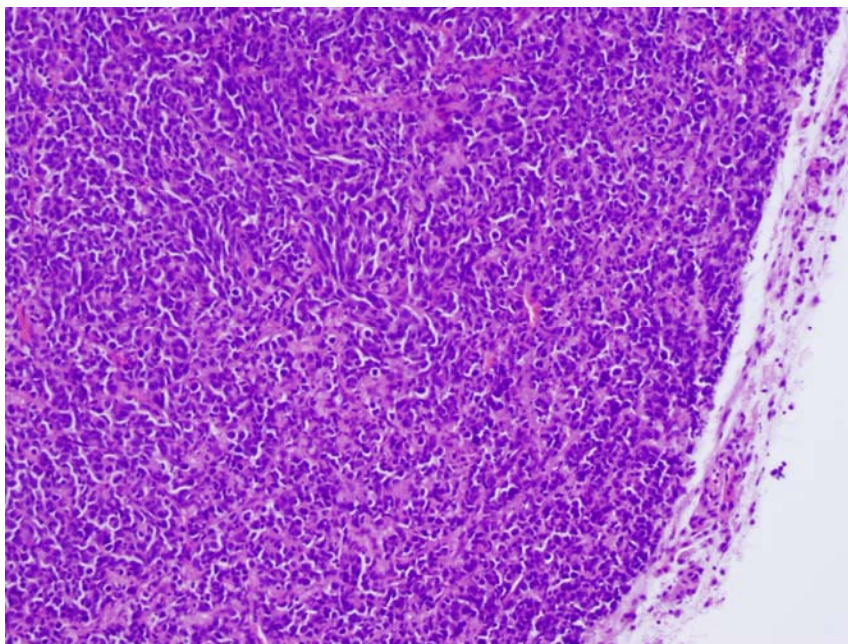

Supplement: Supplementary Figure 4 [file 6605668x4.pdf]
