# Supplementary material for: Molecular characterisation of side population cells with cancer stem cell-like characteristics in small-cell lung cancer
Source: Br J Cancer. 2010 Apr 27;102(11):1636–44. doi: 10.1038/sj.bjc.6605668 (PMC2883147; doi:10.1038/sj.bjc.6605668)
Supplement: Supplementary Figure 3 [file 6605668x3.pdf]

Supplemental Figure 3

A

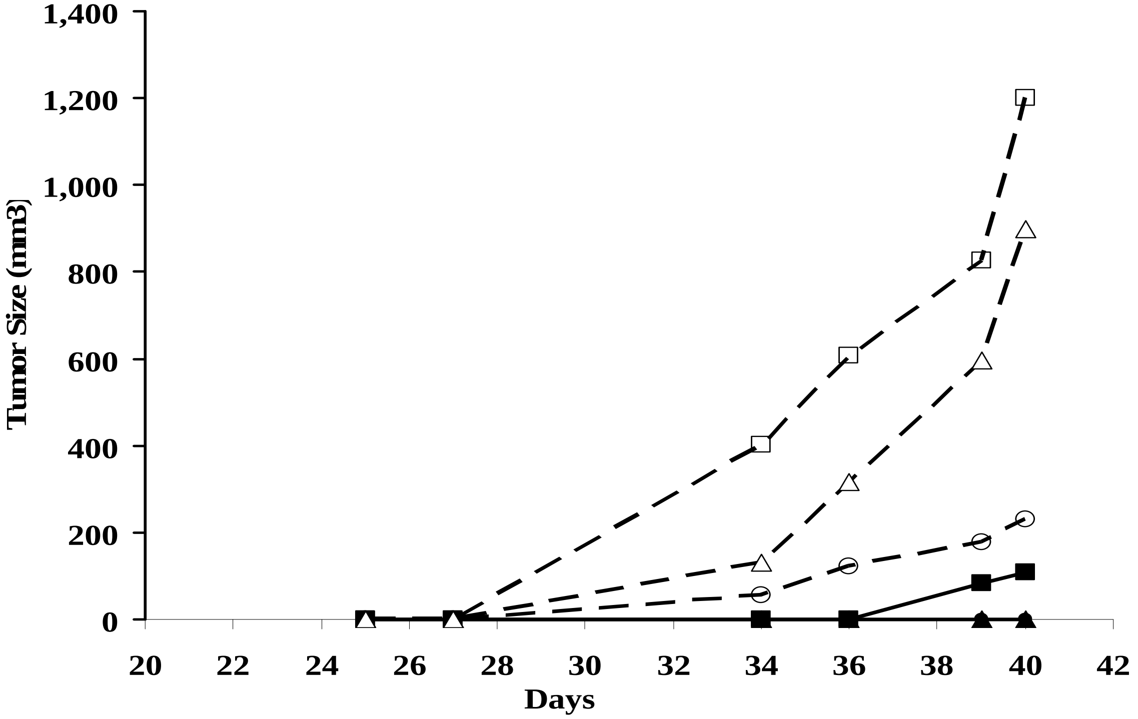

B

Number of mice with H526 tumors/number of xenografts (%)

Cell Number	SP Fraction	Non-SP Fraction	P value*
50	4/7 (57)	0/3 (0)	0.19
100	7/7 (100)	1/8 (13)	0.001
500	7/9 (78)	5/9 (56)	0.37
1000	5/6 (83)	2/8 (25)	0.1

* Determined by two-sided Fisher's exact test.
