# Supplementary material for: Molecular characterisation of side population cells with cancer stem cell-like characteristics in small-cell lung cancer
Source: Br J Cancer. 2010 Apr 27;102(11):1636–44. doi: 10.1038/sj.bjc.6605668 (PMC2883147; doi:10.1038/sj.bjc.6605668)
Supplement: Supplementary Table I [file 6605668x5.pdf]

Supplemental Table I

	H146		H526	
	**SP**	**Non-SP**	**SP**	**Non-SP**
**SP/Non-SP**	**0.8+/-0.1**	**98.1+/-0.4**	**0.9+/-0.4**	**96.3+/-1.8**
**CD56**	**93+/-3.0**	**99.3+/-0.2**	**82.6+/-8.8**	**98.8+/-0.1**
**CD90**	**96+/-1.3**	**98.7+/-1.0**	**88.5+/-3.0**	**99.2+/-0.2**
