# Supplementary material for: Molecular characterisation of side population cells with cancer stem cell-like characteristics in small-cell lung cancer
Source: Br J Cancer. 2010 Apr 27;102(11):1636–44. doi: 10.1038/sj.bjc.6605668 (PMC2883147; doi:10.1038/sj.bjc.6605668)
Supplement: Supplementary Table II [file 6605668x6.pdf]

Supplemental Table II

Genes whose expression differs at least 1.9-fold in SP compared to Non-SP fraction cells.

Gene Symbol	Gene Symbol	Description	Fold Change Ratio *
**1**	**ABCG2**	ATP-binding cassette, sub-family G, member 2	2.7[a]
**2**	**ACTC1**	Actin, alpha, cardiac muscle 1	4.7
**3**	**ADAR**	Adenosine deaminase, RNA-specific	3.1
**4**	**ALPI**	Alkaline phosphatase, intestinal	2
**5**	**APC**	Adenomatous polyposis coli	3.9
**6**	**BMP1**	Bone morphogenic protein 1	3.5
**7**	**BMP2**	Bone morphogenic protein 2	3.6
**8**	**CD8A**	CD8 antigen, alpha polypeptide	2.3
**9**	**CD8B1**	CD8 antigen, beta polypeptide 1	3.5
**10**	**CDC2**	Cell division cycle 2	3.2
**11**	**CXCL12**	Chemokine (C-X-C motif) ligand 12	3.1 [a]
**12**	**FGF1**	Fibroblast growth factor 1	3.5
**13**	**GJA1**	Gap junction protein, alpha 1, 43kDa	6.8
**14**	**HDAC2**	Histone deacetylase 2	2.1
**15**	**IGF1**	Insulin-like growth factor 1 (somatomedin C)	2.5 [a]
**16**	**MYC**	V-myc viral oncogene homolog	2.8
**17**	**NEUROG2**	Neurogenin 2	3.2
**18**	**NOTCH2**	Notch homolog 2	2.4
**19**	**SOX1**	SRY (sex determining region Y)-box 1	2.4
**20**	**SOX2**	SRY (sex determining region Y)-box 2	3.4
**21**	**T**	T, brachyury homolog (mouse)	2.1
**22**	**WNT1**	Wingless-type MMTV integration site 1	2.9
**1**	**GJB1**	Gap junction protein, beta 1, 32kDa	-3
**2**	**PARD6A**	Par-6 partitioning defective 6 homolog alpha	-2

* Value average of two independent experiments

[a] Value from one experiment
