# Supplementary material for: Molecular characterisation of side population cells with cancer stem cell-like characteristics in small-cell lung cancer
Source: Br J Cancer. 2010 Apr 27;102(11):1636–44. doi: 10.1038/sj.bjc.6605668 (PMC2883147; doi:10.1038/sj.bjc.6605668)
Supplement: Supplementary Legend [file 6605668x7.doc]

**Supplemental Figures**

**Supplemental Figure 1. Identification of SP fraction in SCLC cells. A:** Identification of SP cells in H146 and H526 cell lines. Cells stained with Hoechst dye were analyzed by flow cytometry as described in Methods. Gated SP fraction was defined as a fraction that decreased following pretreatment with Verapamil as indicated in the panel **B.** Mouse bone marrow cells are shown as positive control on the lower panel. One of more than 4 independent experiments is shown.

**Supplemental Figure 2. Characterization of** non-small cell lung cancer (NSCLC) **cells. A.** Identification of SP fraction in a NSCLC cell line, A549. Cells were stained with Hoechst dye and analyzed by flow cytometry as described in Methods. SP fraction from three independent experiments was 3-5%. Gated SP fraction was defined as a fraction that decreased by pretreatment with Verapamil as indicated on the right. **B.** Detection of ABCG2 (upper panel) and ABCG1 (lower panel) cell surface proteins in SP and Non-SP populations in A549 cells. One of three independent experiments is shown here.

**Supplemental Figure 3.Tumor growth rates of xenografts established using H526 cells.**  Cells sorted from SP and Non-SP fractions were injected subcutaneously into NOD/SCID mice. A. Fifty (circles), 100 (triangles) and 500 (squares) cells were implanted, and tumor growth rates of SP cells (dashed lines, open symbols) and Non-SP fraction cells (solid lines, closed symbols) were monitored biweekly and shown. Average tumor volume was calculated as indicated in Methods. B. Summary of H526 derived tumors formed following implantation of indicated cell numbers from SP and Non-SP fractions. Small tumors that were only palpable at the time when mice had to be sacrificed due to largest volume tumors were scored as positive.

**Supplemental Figure 4. Morphology of H146 xenografts from SP and Non-SP fraction cells.** Tumors generated from 1,000 cells implanted subcutaneously. H&E staining of tumors generated by Non-SP (top panel) and SP cells (bottom panel). The tumors have similar morphology characteristic of SCLC with hyperchomatic nuclei, abundant cytoplasm, finger-like projections, and frequent mitotic figures.

**Supplemental Table I. Decrease in Mean Fluorescence Intensity (MFI) of differentiation markers in SP fraction cells.** SP and Non-SP fractions and fold changes in MFI in H146 and H526 cells for CD56 and CD90 +/- SD from three or more independent experiments.

**Supplemental Table II. Genes associated with normal stem cell biology upregulated in SP fraction of H146 cells detected y SuperArray screen**. Two hundred and fifty ng of total RNA from SP and Non-SP cells was reverse transcribed and cDNA was subjected to real-time PCR as described in Methods. Genes consistently changed in SP cells as compared to Non-SP cells in two independent experiments were normalized to 4 or more housekeeping genes from the same array.
